# Preventing environmental enteric dysfunction through improved water, sanitation and hygiene: an opportunity for stunting reduction in developing countries

**DOI:** 10.1111/mcn.12220

**Published:** 2015-11-06

**Authors:** Mduduzi N. N. Mbuya, Jean H. Humphrey

**Affiliations:** ^1^ Zvitambo Institute for Maternal and Child Health Research Harare Zimbabwe; ^2^ Johns Hopkins Bloomberg School of Public Health Baltimore Maryland 21205 USA

**Keywords:** stunting, nutrition, disease, infant and child nutrition, early growth, sanitation

## Abstract

In 2011, one in every four (26%) children under 5 years of age worldwide was stunted. The realization that most stunting cannot be explained by poor diet or by diarrhoea, nor completely reversed by optimized diet and reduced diarrhoea has led to the hypothesis that a primary underlying cause of stunting is subclinical gut disease. Essentially, ingested microbes set in motion two overlapping and interacting pathways that result in linear growth impairment. Firstly, partial villous atrophy results in a reduced absorptive surface area and loss of digestive enzymes. This in turn results in maldigestion and malabsorption of much needed nutrients. Secondly, microbes and their products make the gut leaky, allowing luminal contents to translocate into systemic circulation. This creates a condition of chronic immune activation, which (i) diverts nutrient resources towards the metabolically expensive business of infection fighting rather than growth; (ii) suppresses the growth hormone‐IGF axis and inhibits bone growth, leading to growth impairment; and (iii) causes further damage to the intestinal mucosa thereby exacerbating the problem. As such, the unhygienic environments in which infants and young children live and grow must contribute to, if not be the overriding cause of, this environmental enteric dysfunction. We suggest that a package of baby‐WASH interventions (sanitation and water improvement, handwashing with soap, ensuring a clean play and infant feeding environment and food hygiene) that interrupt specific pathways through which feco‐oral transmission occurs in the first two years of a child's life may be central to global stunting reduction efforts.

## The stunting dilemma

In 2011, 165 million (26%) children under 5 years of age worldwide were stunted as indicated by a height‐for‐age *Z‐*score (HAZ) of −2 or lower (Black *et al.* 2013). This stunting is associated with greater risk of death from infectious diseases in childhood (Caulfield *et al.* 2004; Pelletier *et al.* 1995), poorer cognition, (Grantham‐McGregor *et al.* 2007; Walker *et al.* 2011) poorer educational outcomes (Alderman *et al.* 2006; Maluccio *et al.* 2009) and lower adult earnings (Hoddinott *et al.* 2013). For these compelling reasons, normalizing child growth during the window of opportunity – between conception and the first two years of postnatal life (Victora *et al.* 2010) – represents an important long‐term investment.

Analyses of the effects of improved dietary intake on child growth suggest that a nutritionally adequate diet is necessary but not sufficient for ensuring optimal linear growth. A comprehensive review of complementary feeding interventions (Dewey and Adu‐Afarwuah, 2008) revealed a growth effect (mean effect size) of 0.0–0.64 length‐for‐age Z‐scores. A comparable linear growth effect (standard mean difference) of 0.08–0.62 length‐for‐age Z‐scores was reported in a recent series of reviews (Bhutta *et al.* 2013; Imdad *et al.* 2011; Lassi *et al.* 2013) of 16 randomized and quasi‐randomized complementary feeding intervention studies. While statistically significant, these analyses demonstrate that the growth effect of the most efficacious of these interventions was +0.7 Z‐scores, which translates to a modest 30% reduction in stunting because the average linear growth deficit is −2.0 HAZ by 24 months among African and Asian children (Victora *et al.* 2010).

Similarly, disease explains only a part of the variation in stunting. Infection has long been understood to be central to the interactive relationship between disease and nutrition (Scrimshaw *et al.* 1968), with infectious disease episodes increasing the risk of a child being undernourished and vice versa. However, the association between diarrhoea, the most studied and most frequent infection in developing countries, and linear growth is modest, particularly because of catch‐up growth after illness episodes (Briend, 1990; Briend *et al.* 1990). In a pooled analysis of nine studies, a higher cumulative burden of diarrhoea before 24 months was associated with greater odds of being stunted at 24 months (Checkley *et al.* 2008). Additional analyses of seven of these studies (Richard *et al.* 2013; Richard *et al.* 2014) revealed that the association between diarrhoea burden and linear growth is small (0.38 cm, which translates to about 1/15th of the average height deficit at 2 years of age among African and Asian children), and provided further evidence of catch‐up growth: when diarrhoeal episodes were followed by diarrhoea‐free periods in the first two years of life, catch‐up growth allowed children to regain their initial trajectories. The findings of these analyses are consistent with other observations that reductions in clinic presentations of diarrhoea were not associated by improvements in nutritional status (Poskitt *et al.* 1999); and the introduction of a highly effective programme to treat infectious diseases dramatically reduced infant mortality but had no effect on growth (Rousham and Gracey, 1997).

**Key messages**

- The recalcitrance of stunting to diet and disease control interventions has led to the hypothesis that a primary underlying cause of stunting is subclinical gut disease (EED).
- In the context of marginal diets and recurrent infections, EED likely explains a significant portion of the unresolved stunting affecting one in every three children in developing countries.
- Avoiding ingestion of enteric pathogens and other causative microbes by infants and young children could preventmost of the EED burden.
- Interventions aimed at preventing and reducing EED, particularly through babyߚtargetedWASH interventions, may be critical to global stunting reduction efforts.

The realization that most stunting cannot be explained by poor diet or by diarrhoea, nor completely reversed by optimized diet and reduced diarrhoea has led researchers to reexamine papers published over the past several decades that have posited a linkage between unsanitary living environments leading to an acquired asymptomatic but chronic gut injury with systemic immunostimulation and poor growth (Rosenberg and Solomons, 1978; Rosenberg *et al.* 1974; Solomons, 2003; Solomons *et al.* 1993). Frequently, researchers have assumed that any growth benefits of WASH interventions are mediated through reduced diarrhoea. Accordingly, because the linkage between diarrhoea and growth is so weak, the 2008 Lancet Nutrition series estimated that WASH interventions implemented at scale would reduce stunting by only 2.5%, when they modelled the effect through diarrhoea. Other observations suggest the effect of WASH on linear growth may be independent of diarrhoea: in a cross‐sectional analysis of DHS data from eight countries, Esrey (1996) noted that optimum water and sanitation were more strongly associated with linear growth than it was with diarrhoea. In a longitudinal cohort of Gambian children from age 8 to 64 weeks, measures of chronic immunostimulation were highly correlated with measures of enteropathy, and together were strongly predictive of poor linear growth (Campbell *et al.* 2003) further strengthening the hypothesized role of enteropathy and immunostimulation in stunting. Solomons draws parallels between the conditions of these infants and animal husbandry (Solomons, 2003; Solomons *et al.* 1993); noting that either cleaning up the environmental conditions for chickens or adding antibiotics to pig fodder can improve growth and enhance meat production, he presents a major clue that the cause of enteropathy is environmental.

Humphrey (2009) integrated this longstanding literature into a hypothesis that exposure to poor sanitation and hygiene causes this enteropathy, now termed environmental enteric dysfunction (EED; Keusch *et al.* 2013) and that this EED (rather than diarrhoea) is the primary causal pathway from poor sanitation and hygiene to stunting. A more recent observational study supports this hypothesis: Bangladeshi children living in environmentally clean households had less severe EED and higher HAZ than children from contaminated households (Lin *et al.* 2013).

## Environmental enteric dysfunction as a modulator of growth

### The healthy gut

**Figure 1:**
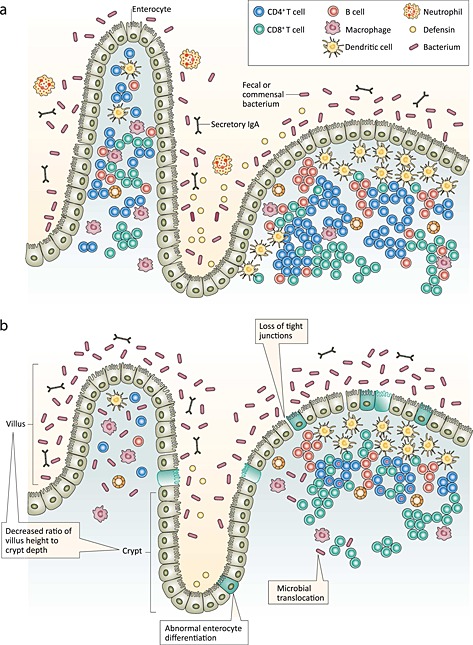
The intestinal epithelium in health (a) and with environmental enteric dysfunction (b). Adapted and reprinted with permission from Macmillan Publishers Ltd: *Nature Reviews Microbiology:* Sandler and Douek (2012).

The healthy small intestine is a complex organ consisting of multiple functional elements: a mucus layer containing defensins and immunoglobulins, a single layer of epithelial cells sealed by tight junctions, and the lamina propia and submucosa containing immune cells (Hodin and Matthews, 2008; McKay *et al.* 2010). The intestinal epithelium, illustrated in Fig. 1a [adapted from (Sandler and Douek, 2012)] forms a one‐cell‐thick interface between the internal organism and external luminal environment. It also comprises two major compartments, the villus and the crypt, which play major roles in the digestion and absorption of nutrients, in absorption and secretion of water and electrolytes and in intestinal immune function (Hodin and Matthews, 2008; Jaladanki and Wang, 2011; Peterson and Artis, 2014). The villous compartment comprises mature, absorptive cells that form finger‐like projections extending into the intestinal lumen thereby amplifying the absorptive surface area 20‐fold. The crypt is a contiguous pocket of epithelial cells at the base of the villus that is populated by younger epithelial cells involved primarily in secretion of antimicrobial proteins and stem cells that continually divide and migrate towards the villous surface. Collectively, these features are responsible for the barrier, immune and absorptive functions of the small intestine.

### The ‘impoverished’ gut: environmental enteric dysfunction

#### What is it?

Environmental enteric dysfunction is a subclinical disorder of the small intestine that is characterized by villous atrophy, crypt elongation, inflammatory cells infiltrating the crypts and a loss of barrier function or increased permeability (Keusch *et al.* 2014; Prendergast and Kelly, 2012). The term refers to a phenomenon of impaired intestinal function rather than a clinical syndrome or entity with diagnostic criteria (McKay *et al.* 2010). The nomenclature for this subclinical condition has evolved with improved understanding from tropical enteropathy to environmental (acquired) enteropathy and is currently referred to as EED (to focus on the functional alterations) (Keusch *et al.* 2013).

#### Epidemiology

Initially described as a condition of the tropics (Desai *et al.* 1969), enteropathy is virtually ubiquitous among persons living in conditions of poverty (Baker, 1976; Keusch, 1972; Menzies *et al.* 1999). Any explanation of the ubiquity of this condition in developing countries must take into account the observation that the intestinal morphology of stillborn fetuses and newborns in these contexts are normal, thereby demonstrating that the disorder is acquired and not genetic (Cook *et al.* 1969; Stanfield *et al.* 1965). South Asian adults with this condition and Peace Corps volunteers who had spent some time in such environments were observed to ‘recover’ when they spent a duration of time away (Gerson *et al.* 1971; Lindenbaum *et al.* 1971), suggesting that the causative factor was environmental. However, recovery rates between these groups differ: South Asian adults who migrated to Europe or the United States had enteropathy on arrival, which resolved within ~5 years (Gerson *et al.* 1971), while asymptomatic American soldiers in Vietnam (Sheehy *et al.* 1968) and Peace Corps volunteers in Pakistan (Lindenbaum *et al.* 1971) who acquired enteropathy within a few month's residence in these settings recovered in the course of 4–5 months after returning to the United States. This suggests that enteropathy is reversible, but that recovery is relatively slow, especially among people who have lived in an unsanitary environment and presumably had the condition throughout their lifetime.

#### Aetiology

Although the pathogenesis of EED is unclear, it has been linked to environmental contamination in general and faecal contamination in particular (Baker, 1976; Lindenbaum *et al.* 1972) and likely represents an adaptive response to a contaminated environment. In response to a prolonged and persistent exposure to enteric pathogens and enterotoxins expressed by pathogenic bacteria, intestinal morphology is altered in a number of ways; the most frequently observed being crypt hyperplasia and villous atrophy. In EED, illustrated in Fig. 1b, crypts are elongated with rapidly increased cell production rate (Cook *et al.* 1969; Veitch *et al.* 2001). This crypt hyperplasia is etiologically related to partial villous atrophy (Desai *et al.* 1969; Fagundes‐Neto *et al.* 1984) or shortening, fusing and broadening of the villi – resulting in the architecture of the gut becoming flatter and blunted with an appearance of leaves and ridges rather than the typical finger‐like projections (Haghighi and Wolf, 1997; Prendergast and Kelly, 2012). The main functional implication of these changes in intestinal architecture is reduced absorptive capacity secondary to the diminished surface area. Because absorptive cells are concentrated in the villous section, shorter villi and deeper crypts have fewer absorptive, and more secretory, cells further compromising nutrient absorption (Nabuurs *et al.* 1993).

At the cellular level, hyperstimulation of enteric T‐cells appears to be important in the pathogenesis of EED (Veitch *et al.* 2001). In a human fetal intestinal explant model, a cell‐mediated immune response was elicited by stimulating T‐cells resulting in a 10‐fold increase in the rate of crypt epithelial cell proliferation and subsequently shorter villi, demonstrating that activation of T‐cells is important in the pathogenesis of EED and that crypt hyperplasia precedes villous atrophy (Ferreira *et al.* 1990). In similar studies of induced T‐cell hypersensitivity (Lionetti *et al.* 1993; MacDonald and Spencer, 1988), some explants showed villous atrophy and crypt hyperplasia, whereas in others, there was mucosal damage, which increased in severity with increasing specimen age. This suggests that crypt hyperplasia, villous atrophy and mucosal damage represent a continuum of adaptive to destructive responses to hyperstimulation by abnormally high levels of ingested bacteria.

#### What triggers T‐cell hyperstimulation?

This is the same cellular mechanism underlying inflammatory bowel diseases such as celiac disease or Crohn's disease. However, in EED, these are normal reactions to abnormally high concentrations of bacteria, whereas in these diseases, T‐cells are abnormally hyperreactive to normal stimuli. For example, in celiac patients, this stimulus is gluten, a normal antigen in healthy people, but an abnormal antigen for people with this genetic defect. In impoverished populations, most authors suggest the primary cause is high concentrations of faecal microorganisms. Accordingly, in this paper, we focus on the prevention of EED through reducing exposure to faecal microorganisms. It is important to note, however, that other causative factors may be important in the pathogenesis of EED in some contexts; these include mycotoxin exposure (Smith *et al.* 2012), severe nutritional deficiency (Guerrant *et al.* 2008), human immunodeficiency virus (Kelly *et al.* 1997), and diarrhoea (Behrens *et al.* 1987; Mondal *et al.* 2012). As such, EED prevention in these contexts could require different interventions.

#### Environmental enteric dysfunction is self‐perpetuating!

The intestinal epithelium selectively limits permeation of potentially harmful luminal substances (Hodin and Matthews, 2008). However, certain microbes or bacterial toxins (endotoxins) perturb barrier function either directly through loosening of the tight junctions or by activating various cytokine, neutrophil and proinflammatory mediators (Arrieta *et al.* 2006; Meddings, 2010). This permeable gut allows luminal contents, including microbes (microbial translocation) to cross the epithelial barrier and into systemic circulation (Brenchley and Douek, 2012). Chronic exposure to these insults – among Gambian infants 2–15 months of age (Lunn *et al.* 1991), increased intestinal permeability was observed in 700 out of 922 dual sugar absorption tests (i.e. for 76% of the time) – create a condition of low‐level chronic immune activation.

Worryingly, EED can be self‐perpetuating once it develops, especially if the host and causative agent are not separated (Gerson *et al.* 1971; Lindenbaum *et al.* 1971). Experimental intravenous infusion of bacterial endotoxin administered to healthy humans increased gut permeability (O'Dwyer *et al.* 1988), suggesting a vicious cycle.

### How might environmental enteric dysfunction cause stunting?

Most of the research linking EED and linear growth faltering has been undertaken among rural Gambian infants and young children over the past two decades. In one of their earlier studies (Lunn *et al.* 1991), investigators in the Dunn Nutrition Laboratory monitored infants over a mean of 7.5 months. In addition to the negative correlation between intestinal permeability and monthly length gain (corrected for age), they calculated that impaired intestinal permeability accounted for 43% of linear growth faltering during this period. In a subsequent study (Campbell *et al.* 2003), three different markers of intestinal function (lactulose/mannitol ratio, IgG anti‐endotoxin titers and plasma immunoglobulin concentrations) were individually associated with growth faltering and showed substantial degree of overlap in their relationship with growth. In semi‐partial regression analysis, the three markers were calculated to explain up to 55% of linear growth faltering, suggesting that intestinal permeability, microbial translocation and inflammatory and immune response are all part of a single mechanism that overall predicted up to 55% of the growth faltering observed in Gambian children. This supports the mechanism of translocation of luminal bacteria or bacterial products across a compromised gut mucosa, leading to stimulation of systemic immune/inflammatory processes and subsequent growth impairment. This growth impairment arises in three ways. First, chronic immune activation is metabolically expensive, resulting in the diversion of nutrients to fuel the immune response (Ganeshan and Chawla, 2014). Second, chronic overproduction of proinflammatory cytokines (e.g. interleukin‐6) causes growth impairment that is mediated by a decrease in circulating insulin‐like growth factor (IGF‐1) levels (De Benedetti *et al.* 1997). Third, proinflammatory cytokines (e.g. interleukin‐1; tumour necrosis factor, TNF; interferon gamma, IFNγ) may directly impede linear growth by inhibiting the process of bone remodelling that is required for long bone growth (Bertolini *et al.* 1986; Skerry, 1994; Stephensen, 1999).

In the context of high nutrient requirements (Butte *et al.* 2000; Dewey, 2013) and rapid linear growth in infancy and early childhood (Dewey *et al.* 1992), EED may cause nutrient malabsorption that could further exacerbate its effects on growth. Morphologic data from nutritionally depleted and nondepleted patients suggested that a decrease in villous height is at least partially responsible for the changes in lactulose/mannitol (L/M) ratio, the dual sugar test of absorption and intestinal permeability (van der Hulst *et al.* 1998). Decreased mannitol absorption is a result of a diminished absorptive area, while increased permeation of lactulose may, in theory, be due to a facilitated diffusion of lactulose into the crypt region as a consequence of decreased villous height in addition to permeation due to loosening of the tight junctions. In the Gambian studies (Campbell *et al.* 2002; Campbell *et al.* 2003), the increase in L/M ratio that strongly predicted growth faltering was due to both reduced M and elevated L uptake, indicating that both barrier and absorptive functions of the small intestine were compromised. Furthermore, the loss of enterocyte brush border enzymes required for digestion and absorption (e.g. lactase) that results from villous atrophy can lead to maldigestion and malabsorption (Lunn, 2000).

Recent studies both confirm and provide further insights on these mechanisms. In the Global Enteric Multicenter Study, 83% of children with moderate‐to‐severe diarrhoea seeking care (cases) had at least one pathogen in their stool, as did 72% of matched controls (Kotloff *et al.* 2013) confirming that sub‐Saharan African and south Asian children 0–59 months old are infected with multiple enteric pathogens even when they do not have diarrhoea. In the MAL‐ED study, measures of intestinal inflammation were associated with linear growth faltering, even after controlling for diarrheal illness (Kosek *et al.* 2013) providing longitudinal support for the EED‐stunting pathway. In a case–control study (Prendergast *et al.* 2014) of Zimbabwean infants who were stunted (HAZ < −2; cases) compared with non‐stunted (HAZ > −0.5 controls) at 18 months, an association was observed between stunting and low‐grade inflammation in the first year of life and perturbation of the growth hormone‐IGF axis. Chronic inflammation due to microbial ingestion likely underlies a great deal of the prevalent and intractable stunting in developing countries.

**Figure 2:**
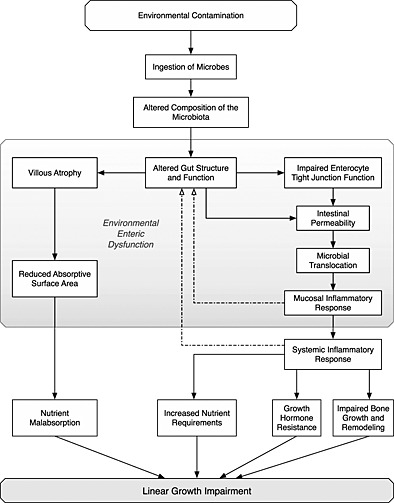
Biological mechanisms linking environmental contamination, environmental enteric dysfunction and linear growth impairment.

The mechanisms linking environmental contamination, EED and stunting are illustrated in Fig. 2. In summary, ingested microbes set in motion two overlapping and interacting pathways that result in a linear growth impairment. Firstly, partial villous atrophy results in a reduced absorptive surface area and loss of digestive enzymes. This in turn results in maldigestion and malabsorption of nutrients. Secondly, microbes and their products impair the barrier function, causing a ‘leaky gut’, that allows luminal contents to translocate into systemic circulation. Chronic exposure to the microbes creates a condition of chronic immune activation, which (i) diverts nutrient resources (that are both scarce and in high demand) towards the metabolically expensive business of infection fighting rather than growth; (ii) suppresses the growth hormone‐IGF axis, and inhibits bone growth and remodelling, leading to growth impairment; and (iii) causes further damage to the intestinal mucosa thereby exacerbating the problem.

There is surprisingly little data in the literature on the relative importance of these pathways (Baker, 1976), especially the malabsorption pathway. We suggest that an effect of malabsorption on growth is plausible only if the amount of nutrients lost in stools is so high that it cannot be compensated by increased food intake. For example, in children, celiac disease can result in stunting of growth without the more classic malabsorptive symptoms of steatorrhoea (Murray, 1999). Also, an effect of infection on growth has been reported during pregnancy (Kayentao *et al.* 2013; Luntamo *et al.* 2013), suggesting a direct effect of inflammation as neither nutrient malabsorption nor poor appetite is likely to play a role during intrauterine life. While it is important to ascertain the full nutritional significance of malabsorption in populations where intake is marginal, the effect of chronic immune activation is likely to be the predominant mechanism.

## Preventing environmental enteric dysfunction

Based on the observational and mechanistic evidence available, poor sanitation and hygiene contribute to, and are possibly the overriding cause of, EED [Lunn, 2000]. Plausibly, avoiding the ingestion of enteric pathogens and any other causative microbes by infants and young children could prevent most of the EED burden.

### Can improvements in WASH prevent or mitigate environmental enteric dysfunction?

The animal literature provides the strongest evidence that cleaning up the environment improves growth. Miller *et al.* (1986) reported increased crypt depth in pigs that were weaned into a ‘dirty’ environment compared with pigs weaned into a ‘clean’ environment. Also, chicks that are raised in environments with faeces, dust and dander (‘dirty chicks’) have higher circulating IL‐1, a major mediator of the immune response, and slower growth than ‘clean chicks’ that are raised in steam‐cleaned cages (Roura *et al.* 1992; Solomons, 2003). This effect is observed even in the absence of pathogenic agents, suggesting an immune response to normally nonpathogenic organisms or other environmental immunogens such as dust and dander. Notably, clean chicks both grow faster and use nutrients more efficiently than dirty chicks (Edwards *et al.* 1960; Roura *et al.* 1992).

Human evidence is limited to observational studies. In a study of Zambian adults (Kelly *et al.* 2004), a hygiene score below the median was independently associated with crypt depth above the median and intestinal permeability (measured by a sugar test of absorption and permeability) above the median. In a recent study (Lin *et al.* 2013), children in environmentally clean Bangladeshi households had lower levels of parasitic infection, less severe EED (lower L : M ratios, −0.32 SD; lower IgG EndoCAb titers, −0.24 SD) and better indicators of attained linear growth (22% points lower stunting prevalence and 0.54 SDs higher HAZ) than children from environmentally contaminated households. In this study, researchers deliberately selected households representing two extremes of the distribution of household environments in order to maximize observed differences, suggesting that it is possible to improve EED and growth within the continuum between these two environmental extremes of rural Bangladesh.

### Which WASH interventions could reduce environmental enteric dysfunction and stunting?

Water, sanitation and hygiene (WASH) interventions have the express objective of preventing the ingestion of harmful microbes by interrupting faecal oral transmission. In recent reviews, Curtis *et al.* (2000) and Brown *et al.* (2013) discuss the variety of these excreta‐related transmission routes, either as a result of direct transmission through contaminated hands or indirect transmission via contamination of drinking water, soil, utensils, food and flies, and acknowledge that the importance of each transmission route varies between pathogens and settings, and that different pathogens are more prevalent in some populations. As such, effective interventions need to address the predominant transmission routes for the target population and context. Specifically, WASH interventions seeking to prevent EED should address specific pathways through which feco‐oral transmission occurs in the first two years of a child's life.

A recent study illustrates the difficulty of aligning interventions with the causal pathways of target problems. Researchers enrolled households in a slum area in Kathmandu, Nepal, and randomly assigned geographic areas to a handwashing promotion intervention (Langford *et al.* 2011). The study reported large reductions in diarrhoea but no improvements in HAZ or EED (mucosal integrity) markers. Additionally, IgG levels rose with age and WAZ and WHZ worsened with age at a faster rate in the intervention group relative to controls. However, there were statistically significant differences between overcrowding and biofuel use in intervention and control areas, which suggests that the poorer health trajectory of children in the intervention group may perhaps reflect conditions of over‐crowding and poverty, leading to greater pathogen exposure through pathways unaffected by handwashing behaviours. The authors suggested in conclusion that ‘for children living in highly contaminated, over‐crowded environments, with poor access to clean water and sanitation, handwashing may be necessary, but not sufficient to reduce levels of subclinical mucosal damage and immune stimulation that are strongly associated with growth faltering’.

In an in‐depth observational study in rural Zimbabwe, infants were found to ingest soil and chicken faeces during exploratory play and mouthing behaviours (Ngure *et al.* 2013; Ngure *et al.* 2014). Using measurements of *Escherischia coli* as a marker, we found that active exploratory ingestion of soil (2100 *E. coli* cfu) and chicken faeces (10 000 000 *E. coli* cfu) posed greater risk of faecal bacteria exposure in terms of microbial load compared with fingers (no *E coli* cfu estimated), food (no *E. coli* cfu estimated) and drinking water (800 *E. coli* cfu). Similar observations of faecal contamination of the play and feeding areas of infants and young children, and ingestion of chicken faeces have been reported in Peruvian slums and Bangladeshi households (Marquis *et al.* 1990). In a recent prospective cohort study of Bangladeshi children, a significant association was observed between caregiver‐reported geophagy and elevated EED disease activity scores (defined as a composite score derived from faecal markers of intestinal inflammation), providing additional evidence that soil might be a direct exposure route for faecal pathogens (George *et al.* 2015). Based on these observations, Table 1 presents a framework for ‘baby‐WASH’ interventions that target the primary feco‐oral microbial transmission pathways among infants and young children. This framework can be used to either guide the formulation of context relevant interventions or as a checklist to ensure that interventions address all relevant pathways through which infants and young children are exposed to and ingest microbes.

**Table 1:** Framework for a package of baby‐WASH interventions to interrupt feco‐oral transmission in the first two years of life

Intervention objective	Timing	Hardware – inputs		Software – behaviour change messages	
		Access (provision, demand creation)	Practical/technical considerations	Utilization (encouragement, demand creation)	Triggers/motivators
Reduce faecal load in living environment	Always	Household sanitary facility (toilet).	Preferably one that facilitates or ensures fly control.	Use of sanitary facilities by all household members. Safe disposal of child faeces.	Disgust has been shown to be an effective trigger for behaviour change.
Reduce faecal transmission via hands	Always	Handwashing facility, soap/scrubbing agent, water (quantity)	Placement of the handwashing facility – (visual) cue to behaviour. Availability of soap or other scrubbing agent (e.g. ash) near handwashing facility.	Handwashing with soap by all household members (including children) at key potential contamination events (e.g. after faecal contact, before handling food and before feeding)	Disgust is also effective in triggering hand washing.
Exclusive breastfeeding	First 6 months	N/A	N/A	Breastfeeding only, to the exclusion of non‐breastmilk items fed for either nutritive or protective (prevention or treatment of perceived childhood illnesses)	Nurture, with a focus on protecting children from potentially harmful non‐breastmilk liquids, foods and traditional remedies
Improvement of drinking water quality	6 months (after 6 months EBF)	Safe water source. Drinking water storage containers. Treatment agent/model (e.g. solar, chlorine) at the point of use.	Water treatment agent should meet organoleptic (taste and smell) expectations of household members.	Water treatment at the point of use. Drinking of treated water by all household members.	Associated taste and smell of treated water with cleanliness. Nurture is an effective motivator for promoting provision of treated water to children
Avoidance of child faecal ingestion during mouthing and exploratory play (e.g. geophagy, consumption of chicken faeces)	2–4 months (crawling and mouthing)	A clean play and infant feeding environment. (Household improvised or technology, such as a protective play space)	The play space should ensure that the child is protected from contamination while ensuring their developmental needs for exploration and interaction are met. Any benefits should outweigh technical and sociocultural burdens of any new technology introduced.	Awareness of risks associated with playing in an environmentally contaminated environment, e.g. geophagy, direct/indirect consumption of animal faeces.	Risk awareness. Nurture.
Hygienic handling and preparation of complementary foods	6 months (after 6 months EBF)	N/A	N/A	Hygienic handling and preparation of complementary foods. Provision of freshly prepared foods as much as possible.	Risk awareness. Nurture.
					

Cluster‐randomized trials are currently being conducted to ascertain the effects of WASH and improved nutrition (independently and in combination) on EED and stunting in Zimbabwe (clinicaltrials.gov identifier NCT01824940), Kenya (NCT01704105) and Bangladesh (NCT01590095). These studies will provide much needed causal and mechanistic evidence on the role of WASH in stunting reduction. However, additional complementary questions warrant further research:

- What microbes are important in the aetiology of EED, i.e. commensal vs. pathogenic microbes (Korpe and Petri, 2012)? This question is important in defining the range of environmental microbes and microbial products that interventions should address. Among 40 asymptomatic infants with EED living in an urban Sau Paulo slum, 63% had colonic bacterial proliferation of the small bowel (Fagundes Neto *et al.* 1994). Moreover, because only pathogenic bacteria cause clinical diarrhoea, sanitation/hygiene messages have often particularly focused on avoiding exposure to faeces of young children – the major carriers of these organisms (Jinadu, 2004; Lanata *et al.* 1998). However, it may be that high concentrations of *any* bacteria in the small bowel can cause EED: in experiments comparing germ‐free with conventional animals, commensal bacteria in *low* concentrations exert a trophic effect on the intestinal epithelium shifting morphology from ‘supranormal’ (i.e. very tall villi) to normal, which results in a structurally and functionally competent immune system (Sprinz *et al.* 1961). Thus, high exposure to all faeces (including those from healthy people and animals) may contribute to EED.
- What are the potential roles of antibiotics, probiotics, other anti‐inflammatory agents, and changes in the composition of the microbiome in treating and preventing EED (Petri *et al.* 2014)? These preventive and therapeutic options for EED represent an area of active research (Galpin *et al.* 2005; Jones *et al.* 2014; Ryan *et al.* 2014; Trehan *et al.* 2009) and discussion (Petri *et al.* 2014; Prendergast and Humphrey, 2014), as we simultaneously seek to better understand the pathogenesis, mitigation and measurement of EED.
- What is the role of maternal EED on linear growth faltering that occurs *in utero*? Approximately 25% of the stunting observed at 2 years occurs *in utero*, i.e. born shorter than they should be: as such, understanding the contribution of maternal EED would aid the formulation of antenatal, and possibly pre‐conception, interventions. This is being investigated through observational and case–control designs within the SHINE trial in Zimbabwe (NCT01824940).
- Which biomarker or biomarkers of EED should be used as (i) normative standards and cutoff points for ‘*counting* the affected’; (ii) indicators of risk for *screening* populations and targeting interventions; and (iii) indicators for *measuring response*(*s*) to interventions (Habicht and Stoltzfus, 1997; Habicht *et al.* 1982)? No single, validated biomarker of EED is available – currently available biomarkers indicate its structural and functional characteristics relative to the interacting and overlapping processes illustrated in Fig. 2. The process of identifying effective biomarkers and defining measurement protocols continues to evolve with heightened interest in the causes, pathogenesis, effects and responsiveness, of EED (Guerrant *et al.* 2013; Keusch *et al.* 2013; Keusch *et al.* 2014; Korpe and Petri, 2012; Kosek *et al.* 2014; Petri *et al.* 2014). This process would benefit from taking these objectives and applications of the ‘best’ indicator or indicators (Habicht and Pelletier, 1990) into cognizance.

## Conclusion

Chronic exposure to a contaminated environment creates a constant state of survival responses characterized by loss, malabsorption, maldigestion and inefficient utilization of nutrients. In the context of marginal diets and recurrent infections, this ‘impoverished gut’ condition likely explains a significant portion of the unresolved stunting affecting one in every three children in developing countries. To prevent stunting, we need to prevent the onset of EED because (i) EED is self‐perpetuating once it has developed; (ii) recovery from EED is relatively slow even when there is a dramatic change in environment; and (iii) the window for critical growth and development is short (between conception and the first two years of postnatal life). Interventions such as baby‐WASH, aimed at preventing and reducing environmental enteric dysfunction, may be central to global stunting reduction efforts.

## Sources of funding

This publication is based on research funded by the UK Department for International Development/Zimbabwe [AG 201 854–101] and the Bill and Melinda Gates Foundation [OPP1021542].

## Conflicts of interest

The authors declare that they have no conflicts of interest.

## Contributions

MNNM wrote the first draft of the manuscript, which was critically revised by JHH.
